# P-141. Efficacy and Safety of Cefazolin versus Anti-Staphylococcal Penicillins in Methicillin Susceptible Staphylococcus Aureus Endocarditis: A Systematic Review and Meta-analysis

**DOI:** 10.1093/ofid/ofaf695.368

**Published:** 2026-01-11

**Authors:** Sandeep Guntuku, Tejaswini Takkellapati, Zeel Patel, Mounica Vorla, Shanmukha Priya Peddireddy, Vimala Thambi

**Affiliations:** Mamata Medical College, Hyderabad, Telangana, India; Mamata Medical College, Hyderabad, Telangana, India; Maimonides Medical Center, New York, New York; Carle Foundation Hospital, Urbana-Champaign, Illinois; Nuvance Health- Vassar Brothers Medical Center, Poughkepsie, New York; Harlem Hospital Center, Harlem, New York

## Abstract

**Background:**

The existing guidelines recommend cefazolin as an alternative to antistaphylococcal penicillins (ASP) in Methicillin Susceptible Staphylococcus Aureus Infective Endocarditis(MSSA IE), despite lack of comparative studies. There is limited data in this clinical context. This is the first meta-analysis to evaluate the comparative efficacy and safety of these drugs in MSSA IE.

**Figure 1:**
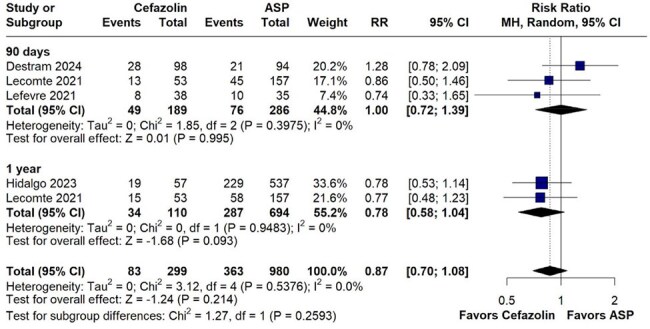
Forest plot comparing all-cause mortality between Cefazolin and ASPs. No significant difference was observed in the overall outcome between cefazolin and ASPs.

**Figure 2:**
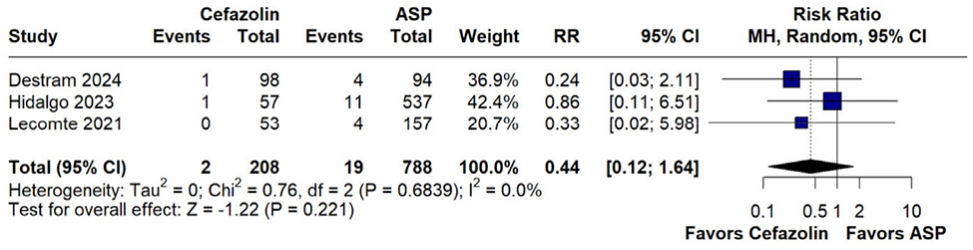
Forest plot comparing relapse rates between Cafezolin and ASP

Relapse was lower in Cefazolin, but the difference was not statistically significant.

**Methods:**

We conducted a systematic search of PubMed, Embase and Cochrane databases to identity studies comparing cefazolin and ASP. The primary outcome consisted of all-cause mortality and the secondary outcomes included relapse rates, embolic events and persistent bacteremia. Pooled risk ratios (RR) with 95% confidence intervals(CI) were calculated using R software 4.4.2 using random-effects model.

**Figure 3.**
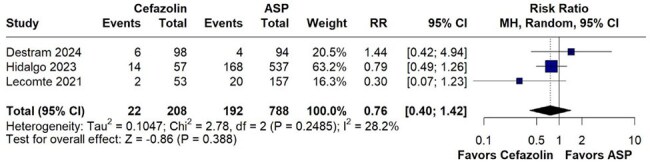
Forest plot comparing embolic event rates between cefazolin and ASPs No statistically significant difference in embolic events was observed between the two groups

**Figure 4.**
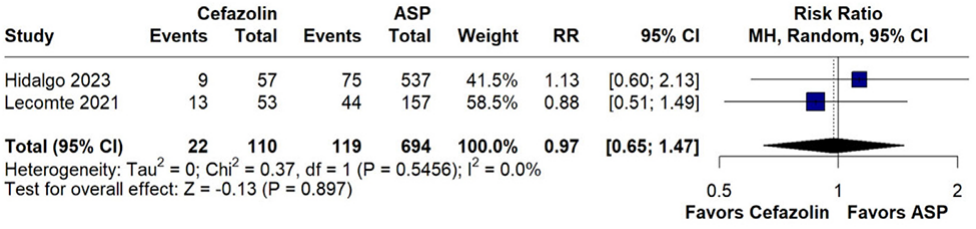
Forest plot comparing rates of persistent bacteremia between cefazolin and ASPs Persistent bacteremia rates were comparable between cefazolin and ASPs.

**Results:**

A total of 4 studies with 1069 were included. No statistically significant difference was observed in all-cause mortality between cefazolin and ASP (RR 0.87, 95% CI 0.70–1.08; p=0.214; I²=0%). Additionally, comparable outcomes were observed in relapse rates (RR 0.44, 95% CI 0.12–1.64; p=0.221; I²=0.0%), embolic events (RR 0.76, 95% CI 0.40–1.42; p=0.388; I²=28.2%), and persistent bacteremia (RR 0.97, 95% CI 0.65–1.47; p=0.897; I²=0%).

**Conclusion:**

Cefazolin demonstrated non-inferior efficacy and safety to antistaphylococcal penicillins for the treatment of MSSA infections, with low heterogeneity across outcomes. These findings support cefazolin as a viable alternative in clinical practice.

**Disclosures:**

All Authors: No reported disclosures

